# Amino acid metabolism by pig intestinal bacteria and consequences for intestinal health and environment: current knowledge and future directions

**DOI:** 10.1186/s40104-026-01491-y

**Published:** 2026-09-07

**Authors:** François Blachier, Mengxuan Tang, Tao Wu

**Affiliations:** 1 Research Unit Nutrition Physiology and Alimentary Behavior, Université Paris-Saclay/AgroParisTech/INRAe, Palaiseau, 91120 France; 2 CAS Key Laboratory of Agro-Ecological Processes in Subtropical Region, Institute of Subtropical Agriculture, Chinese Academy of Sciences, Changsha, 410125 China; 3 Hubei Key Laboratory of Animal Nutrition and Feed Science, Wuhan Polytechnic University, Wuhan, 430023 China

**Keywords:** Amino acids, Bacterial metabolism, Dietary proteins, Green transition, Intestinal epithelium, Intestinal health, Intestinal microbiota, Pig, Pollutant substances

## Abstract

Amino acids available for bacteria within the small intestine of pigs are originating mainly from the dietary and endogenous proteins. These amino acids, in addition to those synthesized by the intestinal bacteria, are used in different metabolic pathways. The amino acids that cannot be synthesized by the intestinal bacteria can be considered as indispensable for them and must thus be obtained from the surrounding luminal fluid. In the pig large intestine, the amino acids used by bacteria originate presumably mainly from proteins not fully digested in the small intestine. In this narrative review, we present the current knowledge concerning the metabolism of amino acids by bacteria found in the pig gut and the effects of the different amino acid-derived bacterial metabolites on the metabolism and physiology of the intestinal microbes. The effects of some among these compounds, either beneficial or deleterious, on the intestinal epithelial cells are then recapitulated. Next, the adverse impact of the emission of two bacterial metabolites derived from amino acids, namely ammonia and hydrogen sulfide, on the environment is described. Future directions in the field of the control of the metabolism of amino acids by the intestinal bacteria for the maintenance of pig intestinal health, as well as for the reduction of the emission of polluting substances are proposed.

## Introduction

The intestinal microbiota, which gathers bacteria, archaea, viruses, and fungi, has been studied in pigs, mainly in terms of composition and, to a lesser extent, in terms of metabolic capacity towards the different substrates [1–4]. The protozoans, although not included as part of the intestinal microbiota, are important members of the pig gut ecosystem [5].

At birth, piglets are suddenly faced with a complex microbial environment that includes bacteria from the maternal vagina and feces, as well as from the rearing environment. The bacterial concentration and diversity in piglet feces increase from birth to weaning [6]. Various dietary components in pig feed have been shown to affect the intestinal microbiota composition [7].

Briefly, the intestinal immune system is continually exposed to antigens and immunomodulatory compounds which are derived from both the diet and the commensal bacteria [8]. The intestine is a major port of entry for many bacterial pathogens, and the intestinal microbiota plays a primary role in the induction, training, and function of the host immune system [9, 10], as well as in protection against colonization by bacterial pathogens [11]. Several metabolites of different chemical structures which are produced by intestinal bacteria have been shown to mediate communication between the commensal bacteria and the host’s immune system [12]. Specific disturbances in the piglet intestinal microbiota composition and/or metabolic activity have been associated with diarrhea and inflammatory processes [13–18].

Several aspects of amino acid metabolism by the intestinal microbiota and their relationships with pig intestinal health have been reviewed in recent years, with emphasis on the effects of amino acid metabolism on microbiota composition in pigs and on the associated growth performance [19–21]. However, these reviews did not include the roles of the amino acid-derived bacterial metabolites for growth and physiology of the intestinal bacteria and of other microorganisms. In addition, the production of polluting compounds from specific amino acids by intestinal bacteria and the adverse roles of these compounds on environment were not considered in these manuscripts.

In this narrative review, we present the sources of amino acids used by the bacteria present in the small and the large intestine of pigs. We detailed the metabolism of amino acids in these intestinal bacteria and relate this metabolism to the physiology and growth of these cells as well as to communication between intestinal microorganisms. Then, we recapitulate how the metabolism of amino acids by the intestinal bacteria can affect the pig intestinal epithelium in terms of physiological functions. Finally, we describe how the metabolic activity of the pig intestinal microbiota can affect the concentration of amino acid-derived polluting substances in the large intestine luminal content and feces.

The search strategy and the literature selection criteria were based on the publications available on PubMed and Google Scholar using several keywords either individually or in combination with no restriction on the time frame. These keywords are the names of the 20 common proteinogenic amino acids, pig, piglet, swine, dietary proteins, amino acid metabolism, intestinal health, intestinal epithelium, intestinal microbiota, bacterial metabolism, amino acid-derived bacterial metabolites, amino acid-derived pollutant substances, ammonia, and hydrogen sulfide. The present review is designed to consider the pig both in terms of its obvious agronomic interest to feed billions of individuals worldwide, and in terms of its utilization as an important experimental model for the study of intestinal physiology.

The last section of this review integrates and synthesizes the different available data, outlines limitations of the studies performed, and proposes future research directions for the study of pig intestinal microbiota in the different areas covered by this review.

## The pig intestinal microbiota composition and global metabolic activity depend on numerous parameters

The composition of bacteria in the luminal fluid of the pig gut during development appears to be influenced by numerous parameters including the gestational stages [22, 23], intrauterine growth status [24], mode of piglet delivery [25], early dietary conditions [26], age of animals, and weaning time [27, 28]. The numbers of microorganisms expand from the small to the large intestine of pigs with microbiota composition differing in the different segments of the intestine [29]. The relatively rapid transit of the luminal fluid in the proximal parts of the small intestine does not allow the development of large concentration of bacteria. In contrast, the concentration of bacteria greatly increases in the distal part of the ileum and increases even further in the large intestine of pigs [30, 31]. The gut microbiota characteristics in pig fecal samples have been determined [32] and the results reveal a total of 7.7 million non-redundant genes representing more than 700 metagenomic species. The meta-analysis of 16S rRNA amplicon sequence data and metagenome-assembled genomes indicate prevalent species within the *Lactobacillus*, *Streptococcus*, *Clostridium*, *Desulfovibrio*, *Enterococcus*, and *Fusobacterium* genera [33].

Part of the bacteria present in the pig intestine is associated with the mucosa, playing an active role against the growth of pathogens [34]. These adherent bacteria are in proximity to the intestinal epithelium [35, 36]. However, quantitatively, most bacteria present in the large intestine luminal content are excreted in feces. This implies a constant and active renewal of the bacterial population in the pig luminal fluid. Such microbial renewal requires presumably intense ATP-dependent anabolic metabolism [37].

## Amino acids originating from alimentary and endogenous host’s proteins are used by the intestinal bacteria

**Fig. 1:**
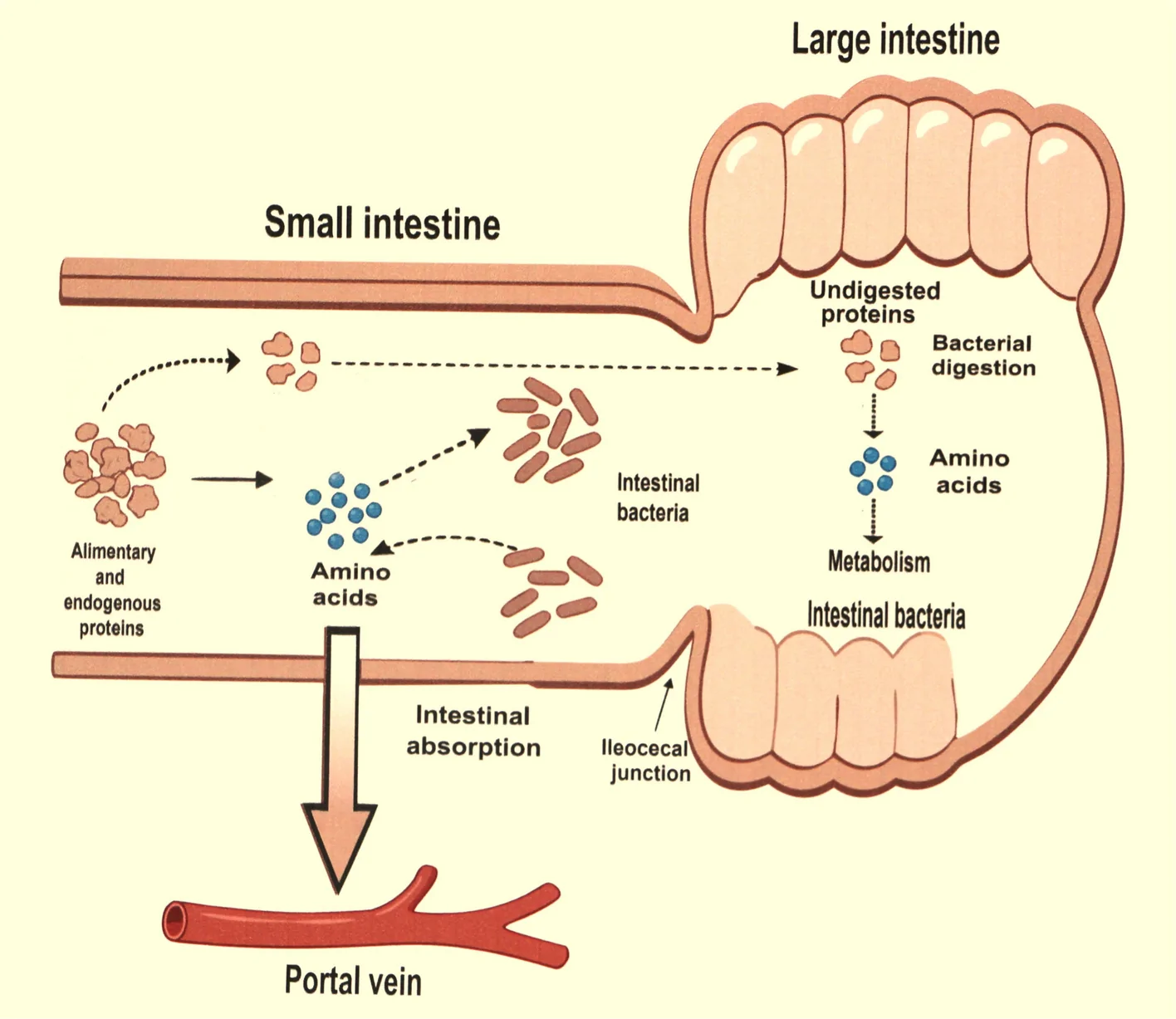
Amino acid metabolism by the bacteria in the small and large intestine. This schematic view represents the digestion of the alimentary and endogenous proteins in the small intestine by the exocrine pancreas proteolytic enzymes. The released amino acids are massively absorbed through the intestinal epithelium in the portal vein while a presumably minor part of amino acids is used by bacteria for their nitrogenous metabolism. Different bacterial species can release amino acids in the luminal fluid after amino acid de novo synthesis. A part of these amino acids can be absorbed through the small intestine epithelium, but the quantity of amino acids supplied by the bacteria to the host remains unknown. A minor part of the undigested alimentary and endogenous proteins is transferred to the large intestine through the ileocecal junction. In the large intestine, these proteins are degraded by the bacterial proteases and peptidases. The amino acids released from this process are not absorbed to any significant extent by the pig large intestine epithelium but can be used by bacteria for nitrogenous metabolism

In the small intestine of pigs, the alimentary and endogenous proteins are degraded by the proteases originating from the exocrine pancreas, thus releasing peptides and amino acids [38]. Several amino acids released from proteins, such as glutamine, glutamate, and arginine, are largely catabolized within pig enterocytes during transfer from the luminal fluid to the portal circulation [39]. The endogenous proteins found in the intestinal luminal fluid include the proteins originating from the exocrine secretions as well as from the exfoliated epithelial cells and mucins released from the epithelium [40, 41]. A minor part of amino acids in the small intestine is believed to be used by the intestinal bacteria, while the bulk of oligopeptides and amino acids released from proteins is efficiently absorbed by the enterocytes and released into the portal vein for utilization in host’s tissues [42] (Fig. 1). Indeed, in pigs, dietary and endogenous host’s proteins are contributors for microbial protein synthesis in the distal ileum [43].

However, the exchange of amino acids between the host and the microbiota in the small intestine may be bidirectional. In fact, some arguments suggest that part of the amino acids synthesized by the small intestine bacteria is released in the luminal fluid and then absorbed through the enterocytes for utilization in the body tissues. For instance, a study determined the incorporation of ^15^N from ^15^NH_4_Cl given orally into lysine in tissues of germ-free rats (without microbes) and in conventional rats (with microbes). These experiments were designed to determine if the ^15^N enrichment found in lysine is compatible with the absorption of lysine synthesized by the intestinal bacteria [44]. The results obtained indicate that the ^15^N-lysine measured in the host tissues is originating from the microbiota metabolic activity. Although no equivalent data were obtained with germ-free pigs, it has been shown that lysine produced by the pig gut microbiota is mainly used for protein synthesis in the pig intestine and liver [45]. Thus, the concept that indispensable amino acids are provided to the host by some bacterial species living within the pig small intestine has been proposed. However, from a quantitative point of view, the amounts of amino acids synthesized by the small intestine bacteria (either anecdotal or significant) which are absorbed and available for the pig tissues remain to be determined, and the relative importance of such a supply for pig growth is still to be documented.

In the large intestine, the situation is quite different since the amino acids available for bacterial metabolism are originating mainly from alimentary and endogenous proteins which have not been fully digested in the small intestine [46]. Although protein digestion in the pig small intestine is an efficacious process, a significant part of undigested proteins (notably those which are more resistant to digestion) moves from the small to the large intestine luminal content through the ileocecal junction [47, 48]. Proteins are degraded into peptides and amino acids by the population of bacteria living in the large intestine, and amino acids are then metabolized by bacteria [49–52]. Amino acids present in the large intestine luminal fluid are presumably mostly used by microorganisms because these molecules are not absorbed to any significant extent by the pig colonic epithelium [53, 54]. However, it is only fair to mention that, to the best of our knowledge, no quantitative studies have estimated the amounts of amino acids provided by the host to the bacteria living within the pig large intestine.

Based on the published data, it is not possible to exclude that some tiny amounts of amino acids are absorbed through the pig large intestine epithelium. In fact, amino acid absorption from microbial origin through the pig large intestine epithelium is measurable based on the appearance of ^15^N-labeled amino acids in the venous blood after infusion of ^15^N-labeled bacteria into the pig cecum [55]. Furthermore, it has been shown that the pig colonic epithelium shows some capacity for amino acid absorption during the neonatal period [56, 57], but this capacity appears transient, being almost lost after this period.

Numerous bacterial species living within the small and large intestine display no metabolic capacity for the synthesis of the 20 common amino acids (and the 2 uncommon amino acids selenocysteine and pyrrolysine) required for protein synthesis and utilization in other metabolic pathways [58–60]. These amino acids have been identified as “bacterially indispensable” for the bacteria concerned, thus implying that they must be supplied from the intestinal luminal fluid to the intestinal bacteria to cover their metabolic and physiological requirements [61]. However, it is important to underline here that most studies aiming at deciphering the metabolism and physiology of the bacteria found in the intestine of pigs have been performed in vitro but not in vivo.

Of note, the amino acids present in the small intestine luminal fluid are, as presented above, mainly originating from alimentary proteins and host’s endogenous proteins, but the supply of amino acids by specific bacterial species for utilization by other bacteria species, even modest, can be envisaged. In fact, some intestinal bacteria like *Escherichia coli* and *Corynebacterium glutamicum* can export their de novo synthesized amino acids into the extracellular medium [62]. These last two bacterial species are members of the pig intestinal microbiota [63, 64]. This can be viewed both as a process of control of amino acid concentration in the producing bacteria and as a process of potential metabolic cooperation between different intestinal bacterial species among the microbial community [65].

Briefly, the studies of amino acid anabolism in intestinal bacteria have been historically focused on a few bacteria species and genera such as *Escherichia coli*, enterotoxigenic *E. coli* (ETEC), enterohemorrhagic *E. coli* (EHEC), *Salmonella*, and *Bacillus subtilis*. ETEC can trigger pronounced diarrhea in piglets [66], while EHEC is well known to provoke severe infection in the large intestine of pigs [67]. Concerning *Salmonella* species, they are pathogens known to provoke enterocolitis in pigs [68].

The description of the different metabolic pathways responsible for the synthesis of the 22 amino acids in bacteria is outside the scope of this review but can be found in recent books and reviews focusing on this subject [58, 60]. Briefly, the diversity of the metabolic capacities to synthesize amino acids in intestinal bacteria can be illustrated by several examples. For instance, *Clostridium perfringens*, known to provoke severe enteritis in newborn piglets [69], lacks genes involved in the synthesis of threonine, serine, glutamate, arginine, histidine, lysine, methionine, as well as aromatic and branched-chain amino acids [70]. Concerning *Lactobacillus johnsonii*, a pig gut commensal [71, 72], this bacterial species is unable to synthesize almost all the amino acids [73]. Other resident intestinal bacteria such as *Campylobacter jejuni* and *Enterococcus faecalis* are not equipped with the whole biosynthetic pathways needed for the synthesis of all amino acids [74]. *Campylobacter jejuni* is a common inhabitant of the pig intestinal tract [75], while *Enterococcus faecalis* is a pig commensal bacterium [76] with reported potential beneficial effects on intestinal villus morphology [77].

Incidentally, it is important to keep in mind that the sole presence of genes involved in amino acid synthesis within the genome of a given bacterial species is insufficient to prove the functionality of the corresponding anabolic pathways. For instance, the genes responsible for the biosynthesis of all the common amino acids have been identified in *Lactococcus lactis*, but still, 6 amino acids, namely glutamate, methionine, isoleucine, valine, leucine, and histidine, need to be supplied from the extracellular medium to allow bacterial growth. *Lactococcus lactis* is a non-invasive and non-pathogenic bacterium in mammals [78] which have recently shown some protective effects in the piglet diarrhea [79]. In addition, *Lactococcus lactis* exerts antimicrobial activity against swine pathogens [80]. In *Lactococcus lactis*, point mutations in genes coding for several enzymes involved in amino acid biosynthesis have been identified, leading to the inability of this bacterium to synthesize several amino acids [81, 82]. Another interesting example of a bacterium unable to synthesize several amino acids (namely cysteine, proline, arginine, valine, and leucine) is *Staphylococcus aureus* [83]. This bacterium is considered as pathogenic in mammals and notably in pigs [84, 85]. Accordingly, this bacterium exhibits an absolute requirement for the five amino acids cited above, despite the presence of genes involved in the different metabolic pathways responsible for the synthesis of these amino acids.

## Amino acids are used by the intestinal bacteria for the synthesis of macromolecules, ATP, and bioactive metabolites

**Fig. 2:**
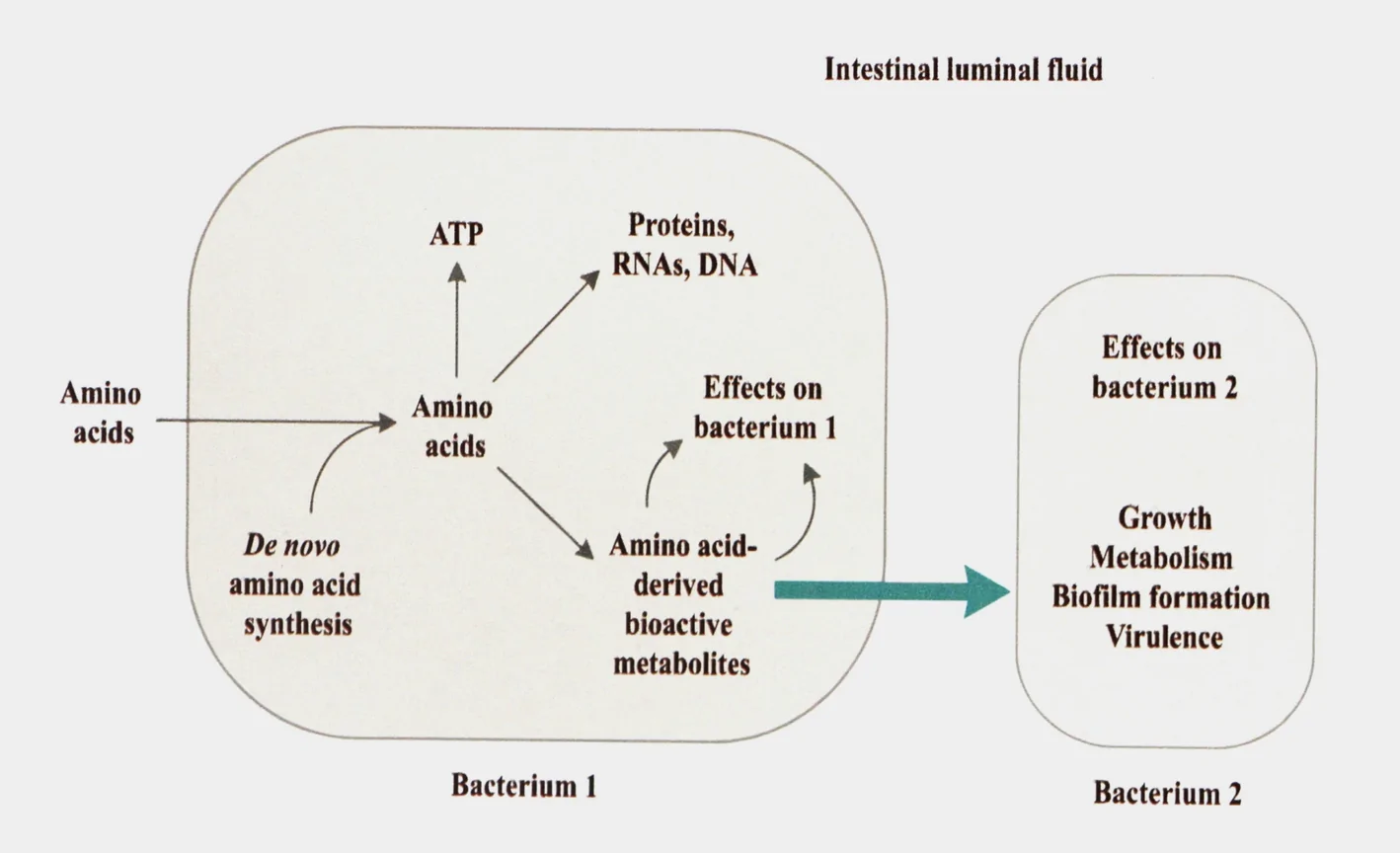
Metabolism of amino acids by the intestinal bacteria. This schematic view represents amino acid metabolism by intestinal bacterium (Bacterium 1). Amino acids are synthesized de novo within bacterium 1 and imported from the intestinal luminal fluid. Amino acids are used within bacterium 1 for the synthesis of proteins, RNAs, and DNA, as well as for ATP production. Amino acid catabolism within bacterium 1 leads to the synthesis of bioactive metabolites. Bioactive metabolites can exert effects on the producing bacteria (Bacterium 1) or/and can be released in the extracellular medium before acting on a given target bacterium (Bacterium 2)

Amino acids are utilized by the intestinal bacteria for the synthesis of macromolecules. These macromolecules include not only proteins but also RNAs and DNA (Fig. 2).

### Amino acids and macromolecule synthesis in intestinal bacteria

Regarding proteins, to face a changing environment within the intestinal luminal fluid (including changes in the nutrient concentration), intestinal bacteria have developed a coordinated protein homeostasis network. Such network includes control processes which modulate both protein synthesis and degradation [86]. These modulation processes have been notably studied in *Escherichia coli* [87, 88]. Regarding the aspect of intracellular protein homeostasis, bacteria can finely tune the translation rate at the 3 major steps, namely initiation, elongation, and termination, thus allowing to maintain a generally stable level of intracellular protein concentration within bacteria even in a changing environment [89].

Regarding RNA and DNA synthesis, 3 specific amino acids, namely glycine, glutamine, and aspartate are precursors for the synthesis of the purine and pyrimidine rings of nucleotides. The proportion of the 3 amino acids devoted to RNA and DNA synthesis in bacteria living in the pig large intestine luminal fluid is likely significant but to the best of our knowledge has not been quantified. Indeed, the number of bacteria within the colonic fluid has been estimated to represent 10^10^ to 10^11^ cells per gram of luminal content [90]. Pyrimidine biosynthesis from amino acids has been studied in bacteria such as *Escherichia coli*, *Salmonella typhimurium*, and *Proteus mirabilis* which are members of the pig intestinal microbiota [91, 92]. The synthesis of pyrimidines needs carbamyl phosphate as one of the required precursors. Carbamyl phosphate is synthesized from glutamine and bicarbonate. Then, carbamyl phosphate and aspartate are precursors for the synthesis of carbamyl aspartate. Carbamyl aspartate is then used for the synthesis in several steps of uridine triphosphate and cytidine triphosphate [93]. The pool of intracellular nucleotides in bacteria is an important parameter which regulates protein synthesis [94], thus indicating complex metabolic relationships to set the rate of protein synthesis in these prokaryotic cells.

### Amino acids and ATP synthesis in intestinal bacteria

Amino acids can be used by bacteria present in the intestinal tract of pigs for ATP synthesis. In growing pigs, only the luminal fluid present in the proximal small intestine is characterized by a higher oxygen concentration when compared with oxygen concentration measured in other parts of the small and large intestine [95]. In fact, these last parts of pig intestine are mostly anaerobic. In such anaerobic conditions, or in the absence of any suitable electron acceptor, strict or facultative anaerobic bacteria found in the pig intestine, such as Clostridia and Fusobacteria [33], can use amino acids as precursors for ATP synthesis. This allows ATP synthesis with a low yield when compared to the yield generally measured in bacteria under aerobic conditions [96]. In *Clostridium* species, including *Clostridium difficile* (found in the pig intestinal tract [97]), typical metabolic pathways, gathered under the name of Stickland reactions, appear notable for ATP production. Stickland reactions are characterized by coupled amino acid oxidation and reduction. One amino acid is acting as an electron donor while another one is acting as an electron acceptor [98]. Electron donors include amino acids such as alanine, leucine, isoleucine, and valine, while electron acceptors include amino acids such as glycine and proline [99]. Stickland reactions in intestinal bacteria equipped for such metabolism allow for the provision of ATP to support bacterial growth notably in situations of short supply of other energy substrates such as carbohydrates [100]. Of note, amino acid utilization for ATP production in bacteria of the pig intestine may be operative in a preferential way when compared to the utilization of other ATP-producing substrates in other metabolic pathways. For instance, *Clostridium stricklandii* is considered as a typical bacterium which preferentially utilizes amino acids such as serine, glycine, arginine, threonine, and cysteine for ATP synthesis [101].

### Amino acids-derived bioactive metabolite synthesis in intestinal bacteria

Numerous intermediary and end products generated during amino acid catabolism in both commensal and pathogenic bacteria present within the pig intestinal luminal fluid have been shown to be active on the metabolism and physiology not only of the producing bacteria but also of other bacteria considered as “receptive bacteria” towards these molecules. Furthermore, emerging data suggest that the synthesis of different metabolites from specific amino acids are involved in communication between some intestinal bacteria and other microorganisms such as intestinal fungi and parasites. In this paragraph, we give typical examples of the individual effects of amino acid-derived bacterial metabolites on the growth, metabolism, and physiology of intestinal microorganisms known to live within the pig gut.

#### Amino acid-derived bacterial metabolites and intestinal bacteria metabolism and growth

**Fig. 3:**
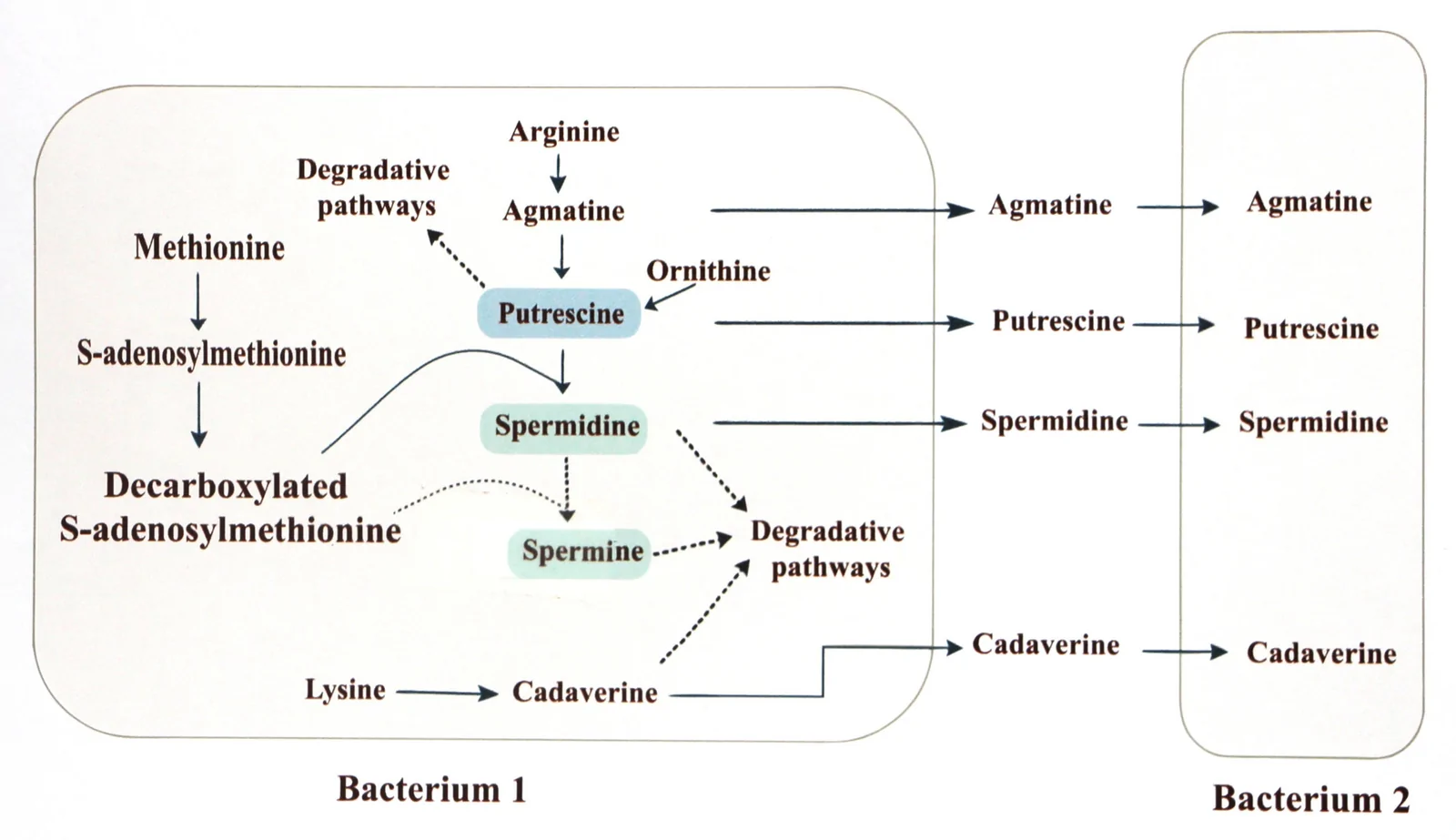
Metabolism of polyamines by the intestinal bacteria. This schematic view represents the synthesis of the different polyamines from the amino acid precursors arginine, methionine, and lysine, as well as from the non-proteinogenic amino acid ornithine. The polyamines can be either degraded or released in the luminal fluid. The concentrations of polyamines within a given bacterium (Bacterium 1) are the net result of endogenous synthesis, import and export from the extracellular medium, and depend also on degradation within bacterium 1. The polyamines released in the intestinal luminal fluid can be captured by another given bacterium (Bacterium 2)

The main polyamines synthesized by bacteria are putrescine, spermidine, agmatine, and cadaverine, while numerous minor polyamine derivatives are synthesized from these main polyamines. For several of these polyamine derivatives, the biochemical pathways involved in their synthesis are generally not operative in eukaryotic cells [102, 103]. The amino acid precursors for polyamine synthesis are arginine, methionine, and lysine as well as the non-proteinogenic amino acid ornithine (Fig. 3).

However, it is worth noting that not all intestinal bacterial species living within the pig gut are equipped with the metabolic machinery necessary for the synthesis of polyamines, thus depending on the import of polyamines present in the intestinal luminal fluid for supply and physiological effects [104]. These polyamines presumably originate from the polyamines present in feed and/or synthesized by other competent bacteria present in the pig intestines which release these molecules in the luminal fluid [105]. Endogenous synthesis of polyamines within pig intestinal bacteria, together with polyamine import and export, as well as degradation in catabolic pathways are the parameters which collectively determine polyamine concentrations in these prokaryotic cells [106]. Putrescine and spermidine are commonly the polyamines found at the highest concentrations in bacteria, followed by agmatine and cadaverine which are found at lower concentrations, and finally by spermine which is rarely detected in most bacterial species where it has been studied [107]. A part of the polyamines present in bacteria is associated with RNA, and such association is related to the important effects of these compounds on the transcriptional and translational processes [104, 108, 109].

Polyamines are involved in intestinal bacteria metabolism. Notably, these compounds can modulate bacterial toxin production and activity. For instance, spermidine increases the production of the bacterial toxin colibactin by colibactin-producing *Escherichia coli* [110]. The other polyamine cadaverine reduces enterotoxin activity by the enterotoxin-producing intestinal bacteria *Shigella* spp. [111]. Among the physiological effects of polyamines, these compounds are well-known to display a generally stimulating effect on the proliferation of the intestinal bacteria, even if this effect is variable depending on the species considered. For instance, *Escherichia coli* made deficient for polyamine synthesis can still grow in media without polyamines, although at a markedly reduced rate, when compared with the wild-type counterpart [112]. This last result indicates that, although not indispensable for the growth of this bacterium, polyamines are required for its optimal growth. Similarly, in the intestinal bacterium *Salmonella enterica*, polyamine depletion reduces but does not abolish the bacterial growth [102]. In contrast, spermidine is critical for *Campylobacter jejuni* growth [113] since the growth of this bacterium is almost suppressed in the absence of this polyamine.

**Fig. 4:**
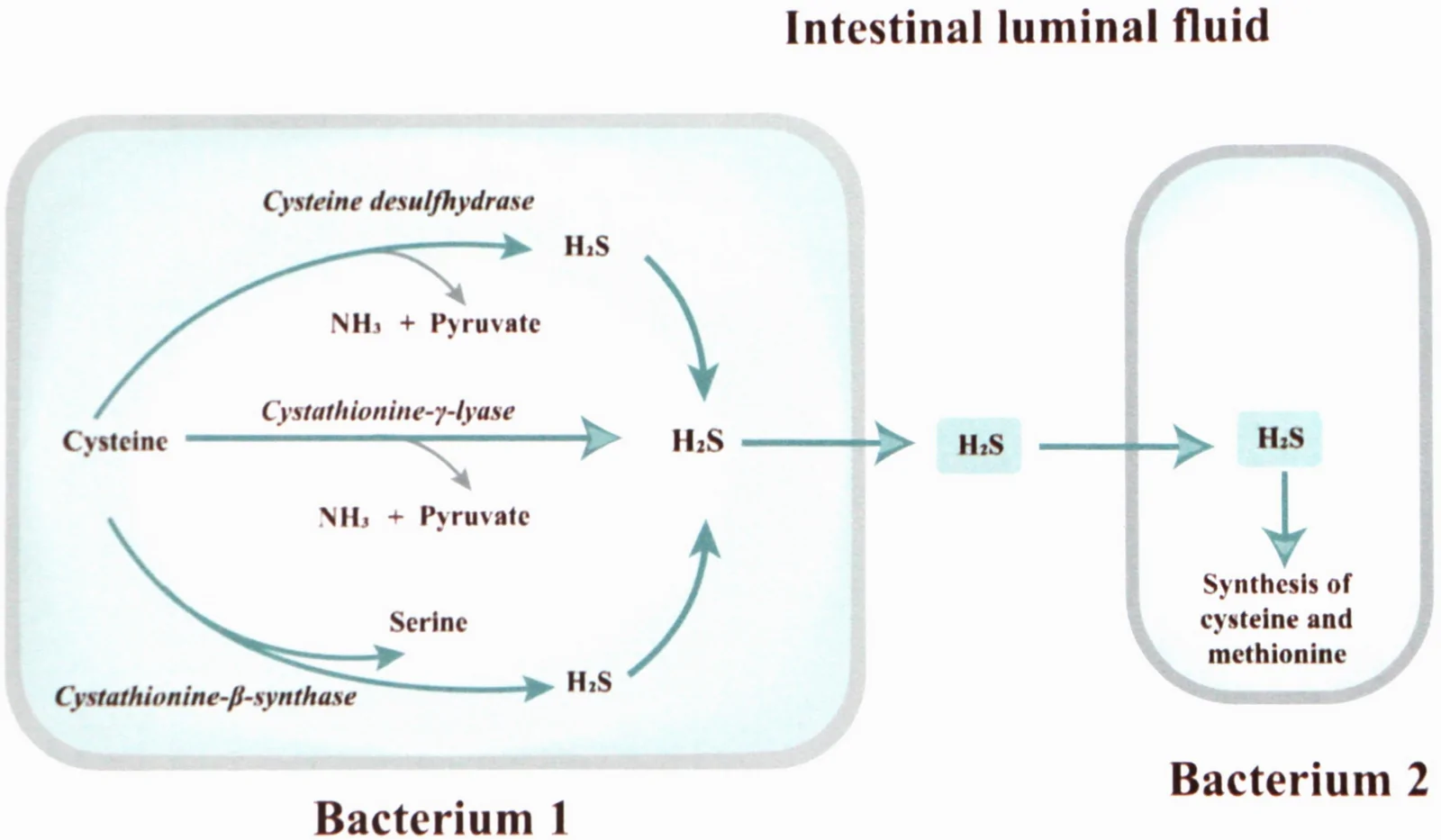
Metabolism of hydrogen sulfide by the intestinal bacteria. This schematic view represents the synthesis of hydrogen sulfide (H_2_S) from cysteine in a producing bacterium (Bacterium 1). H_2_S can diffuse from the producing bacterium to the user bacterium (Bacterium 2). Bacterium 2 can use H_2_S as a precursor for the synthesis of the *S*-containing amino acids cysteine and methionine. Note that the synthesis of H_2_S from sulfate by the sulfate-reducing bacteria is not indicated in this scheme

Hydrogen sulfide (H_2_S) can be synthesized from cysteine by the enzyme cysteine desulfhydrase in pig intestinal bacteria such as *Escherichia coli*, *Salmonella typhimurium* and *Staphylococcus aureus* [114–117]. In addition to this catabolic pathway, H_2_S can be synthesized in bacteria from cysteine through the catalytic action of cystathionine-ɣ-lyase and cystathionine-β-synthase [114] (Fig. 4).

Incidentally, cysteine is not the sole precursor for H_2_S production in intestinal bacteria. Indeed, several bacterial species gathered under the name of sulfate-reducing bacteria found in the pig intestine (such as *Desulfovibrio*) can produce H_2_S from sulfate [33]. H_2_S is a precursor for the synthesis of the S-containing amino acids cysteine and methionine by the intestinal bacteria [60].

H_2_S inhibits the terminal oxidase of the respiratory chain of *Escherichia coli* [118]. However, *Escherichia coli* is equipped with an alternative bd-type oxidase which is not inhibited by H_2_S. The presence of this isoenzyme enables respiration and growth of this bacterium in a H_2_S-rich environment [119].

Nitric oxide (NO) is produced from arginine by nitric oxide synthases present in numerous bacteria including those which inhabit the intestine of pigs such as *Lactobacillus fermentum* [120–122]. Of note, it is important to keep in mind that synthesis of NO from arginine is not the exclusive way for the synthesis of this gasomediator in intestinal bacteria. In fact, intestinal bacteria such as *Campylobacter jejuni* can produce NO through the reduction of nitrite (NO_2_^−^) catalysed by nitrite reductase [123]. NO has been demonstrated to interfere with bacterial growth, notably in intestinal bacteria such as *Salmonella enterica* [124]. The bacteriostatic effect of NO appears to involve the inhibition of enzymatic activities related to bacterial energy metabolism [125], thus suggesting that NO inhibits bacterial growth at least partly through reduction of ATP synthesis.

Indole is produced from the precursor tryptophan by numerous bacterial species including those found in the intestine of pigs like *Escherichia coli* [126]. Indole is efficiently produced by mixed bacterial populations found in pig feces [127]. Indole modulates toxin production by *Klebsiella oxytoca* [128], a bacterium which is a normal resident of the intestine, but which may become pathogenic in specific contexts [129]. In addition to these characteristics, indole has been shown to exert a bacteriostatic effect on intestinal lactic acid bacteria [130].

Dopamine is produced from tyrosine by many bacterial species found in the intestinal luminal fluid such as *Bacillus subtilis*, *Escherichia coli*, *Staphylococcus aureus*, *Proteus vulgaris*, and *Klebsiella pneumoniae* [131]. The bacterial tyrosinases catalyse the conversion of tyrosine to the intermediate dihydroxyphenylalanine (DOPA), this compound being finally converted to dopamine [132].

Dopamine, which is primarily well known to be a neurotransmitter in animals, has been surprisingly shown to stimulate the growth of some intestinal bacteria such as the commensal *Klebsiella pneumoniae* [133]. Such results, as well as those described below, suggest molecular recycling of ancestral compounds active in prokaryotic cells for new functions in eukaryotic organisms [60]. Interestingly, dopamine has been demonstrated to be a siderophore-like iron chelator involved in the optimal growth of *Salmonella enterica* [134]. *Salmonella enterica* is considered as an important zoonotic pathogen which infects the swine intestinal tract [135].

Noradrenaline (norepinephrine) is produced from tyrosine by several intestinal bacteria such as *Bacillus subtilis*, *Escherichia coli*, and *Proteus vulgaris*. Noradrenaline, which acts as a neurotransmitter and hormone in animals, can stimulate the growth of different anaerobic bacteria found in the mammalian intestine such as *Klebsiella pneumoniae*, *Fusobacterium nucleatum*, *Enterobacter cloacae*, *Shigella sonnei*, and *Staphylococcus aureus* [136, 137]. Of note, transferrin, an iron-sequestering molecule, is involved in the effect of noradrenaline on bacterial growth [137].

#### Amino acid-derived bacterial metabolites and intestinal bacteria physiology

Several metabolites produced from amino acids by different intestinal bacterial species living in the pig gut are bioactive not only on the metabolism of bacteria but also on different important aspects of bacterial physiology.

Polyamines are involved in biofilm formation by bacterial species living in the pig gut. Briefly, biofilms are complex structures made by a mixture of bacterial communities embedded in a protective matrix made mainly of bacterial polysaccharides, nucleic acids, proteins, and lipids [138, 139]. Such structures are linked with the modulation of bacterial growth in a changing environment, like the one described in the intestines of mammals [140]. Research on biofilms is urged by the fact that the intestinal pathogens contained within these structures are generally more resistant to antimicrobial agents than same pathogens not protected by biofilms [141].

Potential links between the capacity of pathogenic Enterococci, isolated from the pig intestine and rearing environment, to form biofilm and antibiotic resistance have been documented [142, 143]. Recently, biofilm formation has been shown to contribute to the pathogenicity of *Streptococcus suis*, a bacterial species found in the piglet alimentary tract [144–146].

Polyamines have been recently found to be implicated in biofilm formation. For instance, agmatine is implicated in biofilm formation in the bacterium *Bacillus subtilis* [147] found in the intestine of mammals [148].

Hydrogen sulfide is involved in the regulation of biofilm formation. The role of H_2_S in the formation of colonic intestinal bacteria biofilm formation in relationship with antibiotic resistance has been recently described. Of major importance, the scavenging of H_2_S in several producing bacterial species (including *Escherichia coli*) potentiates both the bactericidal effects of several compounds and disrupts the formation of the bacterial biofilm [149]. This indicates that the endogenous synthesis of H_2_S by these bacterial species is one among important elements for biofilm formation. Further works performed in vivo are necessary to document the relevance of H_2_S binding as a potential strategy for the reduction of antibiotic resistance by some pathogenic intestinal bacteria in pigs.

Beyond the recently discovered stimulating effects of H_2_S on biofilm formation, H_2_S has emerged as a modulator of virulence and susceptibility to antibiotics in the intestinal bacteria *Fusobacterium nucleatum* [150]. Indeed, *Fusobacterium nucleatum*, through its own endogenous production of H_2_S, can modulate its own susceptibility to different antibiotics [150]. In addition, H_2_S appears to act as a protective compound against the action of different antibiotics in the bacteria *Pseudomonas aeruginosa* and *Staphylococcus aureus* [151, 152] which are both found in the intestine. Although the precise mechanisms of action which allow H_2_S to exert its protective effect on bacteria against the action of several antibiotics are not known, some mechanistic elements, using *Escherichia coli* as a bacterial model, have been discovered. In this last bacterium, it has been shown that the sequestration of Fe^2+^ ions by H_2_S, which counteracts the oxidative stress provoked by some specific antibiotics, is presumably involved in the effects of this bacterial metabolite [153]. In the same line of idea, H_2_S is involved in the maintenance of the bacterial redox state in pathogenic *Escherichia coli* strains, an effect which is related to the protection of these bacteria against the oxidative stress provoked by the antibiotic ampicillin [154]. Interestingly, the inhibition of cystathionine-ɣ-lyase, the main enzymatic activity which allows H_2_S synthesis from cysteine in the gut bacteria *Staphylococcus aureus* and *Pseudomonas aeruginosa*, potentiates the efficiency of antibiotics against both bacterial species in different models of infection [155]. These results reinforce the view that H_2_S takes part in the antibiotic resistance described in some intestinal bacterial species. However, the situation is not generalizable to all intestinal bacteria since in *Acinetobacter baumannii* (which can colonize the mammalian gut [156], but which does not produce H_2_S), H_2_S can potentiate the effects of several classes of antibiotics [157]. Thus, the effects of H_2_S, either positive or negative towards the effects of different antibiotics, appear to depend both on the bacterial species examined and on the structure of the antibiotics used making the situation somewhat complicated, notably in the context of pig farming.

Nitric oxide used at low non-toxic concentrations has been shown to increase the dispersal of biofilm macrostructure made by different pathogenic intestinal bacteria such as *Pseudomonas aeruginosa*. The mechanisms at the basis of these effects involve notably NO-sensory proteins [158, 159]. Several NO donors have been tested on different intestinal bacterial species such as *Fusobacterium nucleatum* [160], and anti-biofilm effects of these compounds have been reported [161].

Indole synthesized from tryptophan diminishes the capacity of bacterial cells such as *Listeria monocytogenes* (a bacterium which is occasionally found in the intestine) for motility and aggregation [162]. Of note, this bacterial metabolite diminishes the virulence of intestinal bacterial species such as *Pseudomonas aeruginosa* and *Salmonella enterica* [163, 164].

Skatole (3-methylindole), is produced from tryptophan notably by bacterial species among the *Lactobacillus*, *Clostridium*, and *Bacteroides* genera [165]. Skatole is produced by mixed bacterial populations present in the pig feces [127]. Skatole displays an efficient capacity for the inhibition of biofilm formation by the enterohemorrhagic *Escherichia coli* [166]. Incidentally, from an agronomic perspective, skatole produced in large quantity by the pig intestinal microbiota is well known to be responsible for low organoleptic properties of pork meat [167].

Serotonin (5-hydroxytryptamine), a compound with a function of neurotransmitter in animals, is produced from tryptophan in numerous intestinal bacterial species including *Propionibacterium*, *Lactobacillus*, *Lactococcus*, *Bifidobacterium*, *Streptococcus*, *Bacteroides*, and *Escherichia coli* [136]. Serotonin can regulate the virulence of different bacteria such as *Pseudomonas aeruginosa* in models of rodent infection [168]. However, to the best of our knowledge, serotonin has not been tested in models of pig infected with *Pseudomonas aeruginosa* [169].

Noradrenaline derived from tyrosine increases the virulence of several anaerobic bacteria, notably of the gut bacterium *Clostridium perfringens* [170, 171].

#### Amino acid-derived bacterial metabolites and protection against adverse conditions

The polyamine cadaverine, which, as previously said, is synthesized from lysine, provides a mechanism of resistance against increased acidity to *Escherichia coli* and the foodborne pathogen *Vibrio parahaemolyticus* which can be recovered in the intestine [172, 173].

Betaine, which is derived from glycine, has been shown to act as an osmo protectant for the intestinal bacteria *Pseudomonas aeruginosa* [174].

Gamma-amino butyric acid (GABA), which is produced from glutamate, is known as the main inhibitory neurotransmitter in animals. GABA is synthesized by simple decarboxylation of glutamate by several bacterial species, including notably intestinal bacteria such as *Lactobacillus* and *Bifidobacterium* [175, 176]. GABA is involved in the tolerance to acidic media of intestinal bacteria such as *Bacteroides* spp. via its participation in the maintenance of the intracellular pH [177, 178].

#### Amino acid-derived bacterial metabolites and competition between intestinal bacteria

The bacterial metabolite *p*-cresol (4-methylphenol), which is synthesized by *Clostridium difficile* from tyrosine, has been shown to give a competitive advantage to this bacterium over other gut bacteria [179]. This result is of major importance when considering that *Clostridium difficile* represents a major cause of colitis and diarrhea in mammals. In fact, this bacterium can cause enteritis and diarrhea in both humans and piglets treated with antibiotics [180–182]. *Clostridium difficile*, which belongs to the cohort of early colonizers in the intestinal tract of piglets, infects these animals in a multifactorial way. The severity of the pathophysiological signs of intestinal infection and the characteristics of disease development is presumably related to several general parameters such as the composition of the maternal milk, the individual intestinal microbiota composition/metabolic activity, and co-infections [183].

The metabolite *p*-cresol can be produced by strict and facultative anaerobes, notably those living in the mammalian intestine [184]. Among these intestinal bacteria, bacterial families and genera like Fusobacteriaceae, Enterobacteriaceae, *Clostridium*, and Coriobacteriaceae, are producing *p*-cresol [185, 186]. As introduced above, the capacity of *Clostridium difficile* to convert tyrosine into *p*-cresol is one central element which gives competitive advantages to this pathogenic bacterium over other gut bacteria such as *Escherichia coli*, *Klebsiella oxytoca*, and *Bacteroides thetaiotaomicron* [179]. By using an experimental rodent model of *Clostridium difficile* infection, it has been demonstrated that by removing the metabolic capacity of *Clostridium difficile* to produce *p*-cresol, this bacterium was less able to recolonize the intestinal tract after an initial episode of infection. As expected, *Clostridium difficile* can tolerate *p*-cresol concentration in the extracellular medium as high as 10 mmol/L [187, 188]. As a matter of comparison, the *p*-cresol concentration measured usually in the pig large intestine averages approximately 1.0 mmol/L [189]. Of note, high-protein diet in piglets increases the *p*-cresol production in the colonic luminal fluid [190].

Succinate is produced as an intermediary or terminal metabolites not only during the catabolism of amino acids within bacteria [191, 192], but also from other substrates such as carbohydrates [193, 194]. Succinate produced by the gut microbiota promotes *Clostridium difficile* growth in a rodent model of infection by this opportunistic pathogen [195]. Interestingly, *Clostridium butyricum*, an intestinal bacterium which produces (among other compounds) the short-chain fatty acid butyrate, is found in the intestine of mammals including pigs [196]. This bacterium diminishes the growth of *Clostridium difficile* in a rodent model infected by this intestinal pathogen [197]. The effect of *Clostridium butyricum* is associated with a decreased concentration of succinate in the large intestine luminal fluid. However, no direct causal relationships between the diminution of succinate concentration and the diminution of *Clostridium difficile* proliferation have been demonstrated. Whatever the mechanisms at the basis of the effects of succinate, the decreased concentration of this metabolite is apparently the net result of the overall metabolic activity of the intestinal microbiota. Of note, *Clostridium butyricum*, independently from its inhibiting effect on the growth of *Clostridium difficile,* has been recently suggested to exert beneficial effects on the pig gut. In fact, when *Clostridium butyricum* is used as a feed additive given to sows, it reduces diarrhea incidence in piglets [198]. Furthermore, dietary supplementation with *Clostridium butyricum* in weaned piglets apparently exerts positive effects on intestinal morphology and barrier function [199, 200].

#### Amino acid-derived bacterial metabolites and communication with other intestinal microorganisms

The emerging field of research devoted to the communication between intestinal bacteria and other intestinal microorganisms is of peculiar importance. This is because it can presumably provide a better understanding of the mechanisms which regulate the heterogeneous composition of the microbial community within the mammalian intestine.

Indole synthesized by intestinal bacteria from tryptophan diminishes the virulence and growth of the fungal species *Candida albicans* [201]. This indicates a potential means of communication between intestinal bacteria and a common fungus in the mammalian intestine.

Oxaloacetate is synthesized in bacteria as an intermediate in the tricarboxylic acid cycle during the catabolism of amino acids and carbohydrates. The production of oxaloacetate by *Escherichia coli* increases the survival of the amoeba parasite *Entamoeba histolytica* in the large intestine luminal fluid [202], thus indicating potential bacteria-parasite communication within the mammalian intestine. Although pigs are generally not host for *Entamoeba histolytica* [203], low occurrence of this parasite in the pig gut has been reported [204].

## Amino acid-derived bacterial metabolites are involved in the metabolism and physiology of the intestinal epithelial cells

As presented in the previous paragraphs, the synthesis of bacterial metabolites from different amino acid precursors in intestinal bacteria has consequences on the metabolism, growth and physiology not only of the producing bacteria, but also on other bacteria, either commensal or pathogenic.

In addition to these effects, experimental arguments have been reported which indicate that some among the amino acid-derived metabolites exert bioactive effects on the cells that make up the pig intestinal epithelium. Although the studies on this topic are limited, several interesting data have been recently published. In the present paragraphs, we will i. Summarize how the dietary supply given to pigs can modify the production of amino acid-derived metabolites by the intestinal bacteria, ii. Recapitulate the effects, either beneficial or deleterious, of these metabolites on the pig intestinal epithelial cell metabolism and functions, and iii. Present the potential consequences of such effects in terms of intestinal physiology in pigs.

Branched-chain fatty acids which are detected in the pig intestine are produced by bacteria from specific amino acids. The precursors for branched-chain fatty acids are valine, leucine, and isoleucine which give rise to isobutyrate, isovalerate, and 2-methylbutyrate, respectively [205]. Branched-chain fatty acid production within the intestinal luminal fluid can be modified by nutritional intervention. For instance, supplementation with fermented soybean meal reduces isovalerate in the large intestine of piglets [206], while supplementation with chitooligosaccharide or soybean oligosaccharide in mini piglets reduces the isobutyrate and isovalerate concentrations in the colon [207, 208]. Interestingly, supplementation with isobutyrate alleviates diarrhea in weaned piglets [209]. This can be connected to the capacity of isobutyrate to increase the absorption of electrolytes and water through the colonic epithelium [210–212]. Then, these data suggest that compounds present in the pig feed that decrease markedly the luminal concentration of isobutyrate in the pig large intestine may exert counterproductive effects on intestinal water absorption.

**Fig. 5:**
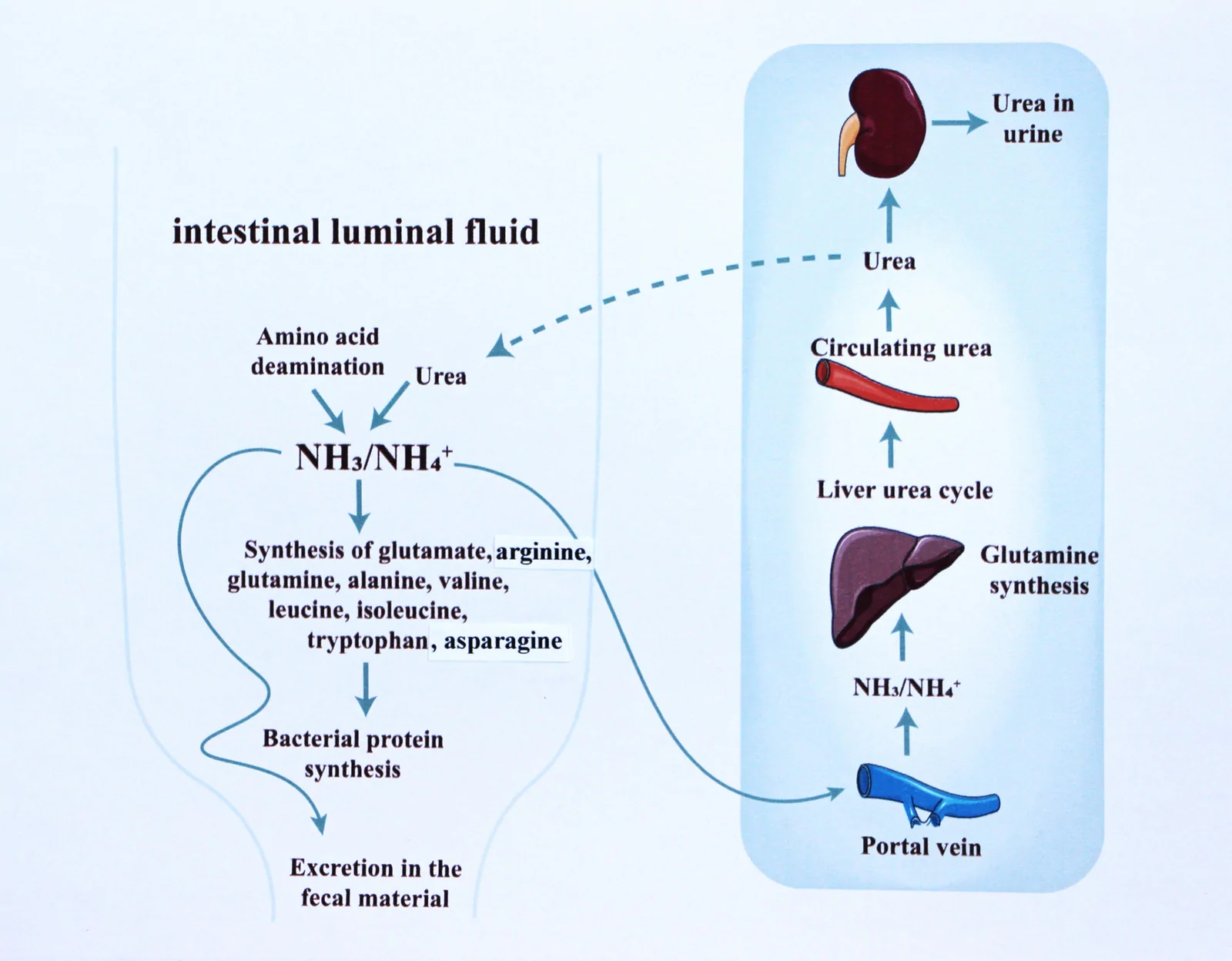
Metabolism of ammonia by the intestinal bacteria. This schematic view represents the production of ammonia (considered as the sum of NH_3_ and NH_4_^+^) by the intestinal bacteria via amino acid deamination and urea degradation. Ammonia is a precursor for the synthesis in bacteria of nine amino acids which can be used for protein synthesis and for other metabolic pathways. Ammonia is efficiently absorbed through the intestinal epithelium into the portal vein. Ammonia is detoxified in the liver urea cycle and is incorporated into glutamate (allowing glutamine synthesis). Although most of the urea produced is excreted into urine, a minor part of urea moves from the blood to the intestinal luminal fluid. Ammonia which is not used by bacteria for amino acid synthesis, and which is not absorbed through the intestinal epithelium, is released in the fecal material

Ammonia (considered as the sum of NH_4_^+^ and NH_3_), is produced by the mammalian intestinal microbiota from amino acid deamination and hydrolysis of urea produced by the host metabolism that moves from blood to the intestinal lumen. Urea hydrolysis is performed by the bacterial ureases [213]. Briefly, ammonia can be both produced and utilized by the community of intestinal bacteria [214]. Regarding ammonia utilization by the intestinal bacteria, this compound is a main source of nitrogen for the synthesis of amino acids such as glutamate, glutamine, arginine, alanine, valine, leucine, isoleucine, asparagine, and tryptophan, as well as other nitrogenous compounds [60]. The concentration of ammonia in the distal part of the large intestine, and thus in pig fecal material, results from bacterial production from nitrogenous precursors, bacterial utilization for amino acids and other nitrogenous compounds, and absorption from the luminal fluid to the portal blood [215–217] (Fig. 5).

The ammonia which is not absorbed and not utilized by the intestinal microbiota is excreted in the feces. Ammonia concentration in the distal colon of pigs fed a high-protein diet is in the 12.1 to 21.0 mmol/L range [218, 219]. Ammonia concentration can be reduced in the colon and caecum of pigs by diminishing the amount of protein in the pig diet, or by replacing part of the dietary proteins with indispensable amino acids in the pig feed [220–222]. By supplementing mini piglets with chitooligosaccharide or soybean oligosaccharide, decreased ammonia concentration in the pig colon is observed [207, 208]. Similarly, dietary supplementation of pigs with wheat arabinoxylan (a soluble fibre) diminishes ammonia concentration in the large intestine [223].

Ammonium chloride, used at a 10 mmol/L concentration, markedly reduces butyrate but not acetate oxidation in pig colonocytes [224]. This result suggests that ammonia in excess adversely affects butyrate oxidation at the steps of activation and/or of beta oxidation. This result is of interest since butyrate is an oxidative substrate, and an ATP-generating substrate, in pig colonocytes [225].

The polyamines spermidine and spermine are components of the sow’s colostrum and milk [226]. Dietary supplementation of piglets with spermine increases the intestinal villus height at weaning [227]. Supplementation with another polyamine (i.e., putrescine) increases the expression of the tight junction protein components ZO-1 and claudin-3 and of the tight junction regulator occludin in the piglet jejunum [228]. Putrescine supplementation in the weaning piglet diet decreases the diarrhea index, and thus the water content in the intestinal luminal fluid [229]. This effect is associated with an increased butyrate concentration in the colonic luminal fluid. Thus, overall, the available data suggest beneficial effects of putrescine and spermine on piglet intestinal physiology.

Indole-related metabolites derived from tryptophan have begun to emerge as bioactive compounds towards the pig intestinal mucosa. Indeed, dietary supplementation with indole-3-carboxaldehyde promotes intestinal epithelial proliferation in weaned piglets, with concomitant upregulation of AhR-related signaling markers, although the effect of this metabolite on barrier function appears limited [230].

While branched-chain fatty acids, ammonia, polyamines and tryptophan-derived metabolites have received most attention, the data regarding the effects of other amino acid-derived bacterial metabolites on the pig intestinal epithelium (namely hydrogen sulfide, nitric oxide, dopamine, noradrenaline, serotonin, gamma-amino butyric acid, *p*-cresol, skatole, betaine, succinate, and oxaloacetate) are scarce. The hydrogen sulfide rapid donor sodium hydrosulfide (NaHS), although being known as an inhibitor of the mitochondrial cytochrome c oxidase activity and of the mitochondrial oxygen consumption in colonocytes [231], does not affect the viability of isolated pig colonic crypt when tested for 4 h at millimolar concentrations [232]. This suggests that an exposure to hydrogen sulfide is not associated with rapid loss of cell viability, maybe because of an efficient H_2_S detoxification system in pig colonic epithelial cells [233]. However, this enzymatic system has not been specifically studied in pig colonocytes.

In the absence of available data from experiments in pigs with the metabolites listed above, we can only refer to data originating from experiments performed with human cells and from experiments performed in rodents. These data, reported below, might be of interest for individuals involved in the pig industry.

Administration of dopamine in the colonic lumen of mice increases water absorption [234]. In addition, dopamine increases mucus secretion in the rat distal colon [235]. Concerning *p*-cresol, it has been demonstrated that this bacterial metabolite diminishes mitochondrial oxygen consumption in human colonocytes, an effect which was paralleled by a decreased proliferation of colonocytes [236]. Furthermore, by using monolayers of human colonocytes, it has been shown that *p*-cresol dose-dependently increases the paracellular transport between colonocytes [237]. This last result suggests alteration of the intestinal barrier function. The effects of succinate on the intestinal epithelial cells are also of interest. In fact, this bacterially derived compound provokes hyperplasia and hypertrophy of goblet cells in the distal small intestine of rodents [238]. Moreover, succinate produced by the intestinal microbiota promotes the expansion of Tuft cells [239]. These cells are secretory cells involved in the production of compounds linked to intestinal immunity. The expansion of Tuft cells is associated with signs of decreased inflammation in the mice model [239].

The results presented in the previous paragraph aim at motivating additional studies in pigs to document the effects (either beneficial or deleterious) of these amino acid-derived bacterial metabolites on the pig small and large intestine epithelium metabolism and physiology.

## Amino acid-derived bacterial metabolites have negative environmental impact: the case of ammonia and hydrogen sulfide

Large-scale pig farming emits numerous pollutants to the local and regional environment including gaseous and particulate matter emissions [240, 241]. Among these polluting substances, the effects of ammonia and H_2_S, synthesized by the pig intestinal bacteria from specific amino acids, have been evaluated from various perspectives. These perspectives include the health of farm workers and neighbouring populations, as well as animal health and agronomic production.

Regarding ammonia, the median emission of NH_3_ in the atmosphere of swine facilities in North America is estimated to be approximately 2.8 kg ammonia per pig and per year [242]. Mitigating ammonia emission from swine manure thus represents a worldwide challenge [243–245].

The ammonia emitted from pig facilities originates primarily from nitrogen excreted by the animals. In pigs slaughtered at 110 kg body weight, it has been assessed that approximately 80% of the excreted nitrogen is present in urine in the form of urea while the remainder is mainly present in the form of NH_3_/NH_4_^+^ in the fecal material [246]. Urea is degraded into NH_3_/NH_4_^+^ by the bacterial urease activities present in the pig feces when urine and feces are mixed. Once released into the environment, ammonia contributes to several detrimental effects. NH_3_ plays a significant role with other pollutants in the formation of atmospheric particles [247]. These particles can exacerbate respiratory issues, notably in agricultural workers [248]. Furthermore, when deposited in water, ammonia in excess can lead to eutrophication thus causing algal bloom and oxygen depletion. Such events are deleterious for different forms of aquatic life [249]. Ammonia in excess in the atmosphere of swine facilities (50 ppm) can exert deleterious effects on the respiratory tract of pigs, inducing signs of lung inflammation, and causing anorexia. These events are associated with alteration of growth performance and economic losses in pig farming [250, 251].

Concerning H_2_S, the median emission from swine facilities represents 0.09 kg/pig/year [242]. The toxicity of H_2_S is mainly related, as previously mentioned, to the capacity of this gas to inhibit the mitochondrial cytochrome c oxidase with an affinity close to that of cyanide [252]. At low levels in the atmosphere, H_2_S acts as a respiratory irritant, while at higher levels, this gas acts as a chemical asphyxiant in both humans and pigs [253, 254]. Thus, H_2_S represents a serious biological hazard for agricultural workers [255].

## Conclusions, limits, and perspectives

The present review recapitulates experimental data which suggest that amino acids derived from alimentary and endogenous proteins play a major role in the renewal of the bacteria present within the intestinal luminal fluid. Such renewal is believed to be necessary to ensure the repletion of the bacteria which are excreted in large quantity in the pig feces every day. This renewal is most likely dependent on intense anabolism with notably active protein synthesis from amino acids synthesized within the different bacterial species and amino acids available from the luminal fluid. All the amino acids that intestinal bacteria cannot synthesize can be considered as indispensable and must then be imported from the luminal fluid for ensuring protein synthesis inside the bacterium. The proteins are not the only macromolecules synthesized from amino acids by the intestinal bacteria, and the purine and pyrimidine rings in DNA and RNAs in growing bacteria are dependent on 3 specific amino acids (glutamine, aspartate, and glycine) for their synthesis. The synthesis of proteins, DNA, and RNAs in intestinal bacteria is dependent on ATP supply. Part of this ATP is produced through amino acid catabolism in bacteria equipped for such amino acid utilization. Finally, amino acids during their catabolism within bacteria produce numerous intermediary and terminal metabolic products, and several of them, when accumulated in significant quantities, have proven to be bioactive in terms of bacterial growth, as well as in terms of bacterial metabolism and physiology (Table 1).

**Table 1:** Bioactive effects of amino acid-derived bacterial metabolites on intestinal bacteria growth, metabolism and physiology

Bacterial metabolites	Amino acid precursors	Modified bacterial parameters
Polyamines	Arg, Met, Lys, Orn	Growth, biofilm formation, toxin production
H_2_S	Cys	Biofilm formation, energy metabolism
NO	Arg	Biofilm formation, energy metabolism
Indole	Trp	Growth, toxin production, virulence
*p*-cresol	Tyr	Competitive advantage
Skatole	Trp	Biofilm formation
Dopamine	Tyr	Growth
Noradrenaline	Tyr	Growth, virulence
Serotonin	Trp	Virulence
Succinate	Several amino acids	Growth

From Table 1, it appears clearly that important aspects of bacterial growth, metabolism and physiology are affected by several bacterial metabolites, thus suggesting complex regulation of these parameters by these compounds. In addition, some amino acid-derived bacterial metabolites such as polyamines, betaine, and gamma-amino butyric acid are involved in the protection of intestinal bacteria against adverse conditions in terms of lower pH and higher osmolarity [172–174, 177, 178]. However, it remains to be demonstrated if such effect is relevant to the changes of pH and osmolarity which can be recorded in the pig intestine in the breeding conditions.

In the field of intestinal bacteria physiology, as presented in this review, important discoveries have been made regarding the implication of amino acid-derived metabolites such as polyamines, H_2_S, NO, and skatole on the modulation of protective biofilm formation [147, 149, 159, 161, 166]. This represents an important field of investigation since biofilm formation and quorum sensing are intimately interconnected [256, 257]. Briefly, quorum sensing is related to the capacity of bacteria to collectively modify their behavior in response to changes in the cell density and species composition within changing local environment [258]. Such changing environment can be clearly documented for bacteria living within the mammalian intestine notably in situation of modified nutritional characteristic [140].

Several studies indicate that metabolites synthesized from amino acids by a producing intestinal bacterium can act both on the producing bacterium itself, but also on other bacterial species, thus suggesting that such communication may represent an important determinant of the bacterial composition within the mammalian intestine. For instance, as detailed in this review, the production of *p*-cresol from tyrosine by the pathogenic bacterium *Clostridium difficile* gives a competitive advantage to this bacterium over other intestinal bacteria [179]. Further works are obviously needed to document in a more thorough manner how the production of active metabolites can influence the respective growth of commensal and pathogenic bacteria within the small and large intestines of pigs.

Emerging data suggest that some bacterial metabolites synthesized from amino acids by some bacterial species within the mammalian intestine are active not only on the producing and target bacteria, but also on other intestinal microorganisms, such as fungi and parasite [201, 202], thus revealing participation of these compounds in the overall composition of the intestinal ecosystem. This aspect remains in its infancy and needs to be further explored and documented because of significant potential applications for pig intestinal health.

However, it is important to underline that the in vivo and in vitro experiments that have been made regarding the effects of bacterial metabolites on bacterial metabolism and physiology have been generally made by using individual compounds. Such experimental situation is not the situation of real life where these compounds are present at the same time in the pig luminal fluid. The situation is complicated by the fact that the amino acid-derived bacterial metabolite concentration in the pig gut may fluctuate according to the nutritional situations [259]. Furthermore, it remains to verify for some bacterial metabolites that the concentrations tested in in vitro and in vivo experiments are within the range of concentrations measured in the intestinal luminal fluid in different situations. From that point of view, it is important to keep in mind that the concentrations of metabolites produced by the intestinal microbiota have been generally measured in the pig feces, and in some cases in the luminal fluid present in the pig large intestine. The fecal concentrations of metabolites represent the concentrations present in the very distal part of the large intestine (i.e., the rectum). Thus, unfortunately, few data are available regarding the concentrations of bacterial metabolites within the different segments of the small and large intestine in pigs. Regarding these last points, porcine intestinal organoid-derived monolayers represent a promising experimental model for testing the direct effects of individual bacterial metabolites and of mixtures of these compounds on the pig intestinal epithelial cells recovered from the different intestinal segments in different situations such as stages of development and alimentary conditions [260].

Apart from the implication of the amino acid-derived bacterial metabolites on the interactions between the intestinal bacteria, some information reported in this review indicate effects of some of them on the pig intestinal epithelium in relationship with its metabolism and physiology. This is the case for instance for ammonia which decreases butyrate oxidation in pig colonocytes [224] and for putrescine which increases the expression of tight junction proteins [228]. Still somewhat little studied, this subject is critical because presumably connected with episodes of diarrhea and inflammation that may occur in piglets [261–263]. Nevertheless, as indicated in the previous paragraph, most of the bacterial metabolites derived from amino acids have been tested individually while increased protein putrefaction within the large intestine of pigs leads to modifications of the concentrations of numerous amino acid-derived metabolites.

Lastly, although having been the subjects of only recent studies, experimental arguments have been presented which suggest that the emission of polluting substances derived from amino acid utilization by the pig intestinal bacteria, such as ammonia and hydrogen sulfide, can be reduced by different means. This is a new and important field of research because these compounds exert obvious adverse effects on the aquatic environment (for ammonia) as well as on human and animal health (for ammonia and H_2_S) [249–255]. Together, ammonia and H_2_S represent two major amino acid-derived gaseous pollutants from pig production, with documented effects on human health, animal welfare, and the broader environment.

Different options can be proposed for a reduction of the emission of these compounds in the environment in a perspective of green transition [264, 265]. First, in pig facilities, as we saw earlier, excretion of ammonia can be decreased by reducing the quantity of crude proteins in the pig feed and/or substituting a part of crude proteins by individual amino acids. For instance, reducing the crude protein level in the pig feed from 20% to 17% and then to 14% results in a decrease of ammonia in all intestinal segments [220], while reduction of crude proteins from 24% to 20% in the pig diet decreased ammonia in the cecum by one third [221]. New studies on that aspect will allow to get additional data on the precise protein requirements for optimal growth and physiology at all stages of piglet development with limited production of polluting substances.

Second, specific indigestible carbohydrates can be adjusted in the pig feed to decrease ammonia production within the intestinal luminal fluid and then in the fecal material. For instance, supplementation of pig feed with different saccharides such as chitooligosaccharide, soybean oligosaccharide or arabinoxylan reduces ammonia in the pig large intestine [207, 208, 223].

Third, there are some reasons to consider that modifications of the pig microbiota composition and/or metabolic activity can reduce the bacterial synthesis of ammonia and hydrogen sulfide. Importantly, ammonia and H_2_S concentrations in the luminal fluid is dependent on their utilization as precursors for the synthesis in intestinal bacteria of different amino acids. Indeed, ammonia is used in bacteria for the synthesis of nine amino acids, while H_2_S is used in bacteria for the synthesis of cysteine and methionine [60]. In other words, it is desirable that ammonia and H_2_S are produced by the bacteria in sufficient quantities to allow sufficient de novo synthesis of amino acids. Such production then participates in the anabolism and renewal of the remaining bacterial population after excretion of bacteria in the fecal material. Nevertheless, such ammonia and H_2_S production should not exceed by much the requirements of these precursors for amino acid synthesis to avoid accumulation of these polluting substances in the intestinal luminal content, and finally in the feces. Last, regarding the emission of H_2_S, such gaseous emission can be reduced by the binding of this compound in the pig intestine and manure with inorganic and organic molecules [266–269].

Integrated studies linking intestinal, fecal, and manure metabolomics with on-farm gaseous emission measurements should be encouraged. Because environmentally relevant pollutants such as ammonia and H_2_S ultimately arise from excreta and manure handling, parallel profiling of amino acid-derived bacterial metabolites in intestinal contents, feces, and manure, together with determination of the microbiota composition and measurements of gaseous emissions in pig production facilities, should help identify signatures associated with higher or lower pollutant output. Such integrative approaches will surely contribute to the development of precision feeding strategies designed at reducing the environmental footprint of pig production while promoting the pork industry.

Finally, progress in the field of pig nutrition, intestinal health, and production performance, as well as in the field of the protection of the environment, is dependent on a constant flow of information originating from additional in vitro and in vivo experiments with amino acid-derived bacterial metabolites. We recommend performing these experiments with bacterial metabolites, tested at their physiological concentrations in media mimicking the intestinal luminal fluid composition, for their effects on the intestinal commensal and pathogenic bacteria found in the pig gut, as well as on the pig intestinal epithelial cells. The effects of nutritional and microbiological interventions on the pig microbiota composition and metabolic activity, and the consequences of such interventions for the defence of the environment and the pig intestinal health in both medium-term and long-term perspective are worth being explored further.

## Data Availability

No datasets were generated or analysed during the current study.

## Abbreviations

ATP: Adenosine triphosphate
H_2_S: Hydrogen sulfide
NH_4_Cl: Ammonium chloride
NO: Nitric oxide
